# Maternal hormone levels among populations at high and low risk of testicular germ cell cancer

**DOI:** 10.1038/sj.bjc.6602545

**Published:** 2005-04-19

**Authors:** Y Zhang, B I Graubard, M A Klebanoff, C Ronckers, F Z Stanczyk, M P Longnecker, K A McGlynn

**Affiliations:** 1 Division of Cancer Epidemiology and Genetics, National Cancer Institute, NIH, DHHS, 6120 Executive Blvd., EPS-7060, Rockville, MD 20852, USA; 2 National Institute of Child Health and Human Development, NIH, DHHS, Rockville, MD, USA; 3 Reproductive Endocrine Research Laboratory, University of Southern California Keck School of Medicine, Los Angeles, CA, USA; 4 National Institute of Environmental Health Sciences, NIH, DHHS, Research Triangle Park, NC, USA

**Keywords:** maternal hormone levels, testicular germ cell cancer, ethnic differences, undescended testes

## Abstract

Ethnic differences in maternal oestrogen levels have been suggested as explaining the significantly higher risk of testicular germ cell tumours (TGCT) of white men than black men in the United States. We therefore examined levels of maternal oestrogens, as well as testosterone and alphafetoprotein (AFP), in 150 black and 150 white mothers in the Collaborative Perinatal Project. Serum levels of estradiol (total, free and bioavailable), estriol, testosterone (total, free and bioavailable), sex hormone binding globulin (SHBG), and AFP were examined during first and third trimesters. We found that the black mothers, rather than the white mothers, had significantly higher estradiol levels in first trimester (*P*=0.05). Black mothers also had significantly higher levels of all testosterone (*P*<0.001) and AFP (*P*<0.001) in both trimesters. In addition, the ratios of sex hormones (estradiol/testosterone) were significantly lower among black mothers. These findings provide little support to the oestrogen hypothesis, but are consistent with higher levels of testosterones and/or AFP being associated with reduced risk of TGCT; alternatively, lower oestrogen/androgen ratios may be associated with reduced risk.

The incidence rate of testicular germ cell tumours (TGCT) among white men in the United States is roughly five times higher than the rate among black men. In addition, the incidence among white men has been increasing during the past several decades (Zheng *et al*, 1996; McGlynn *et al*, 2003; Ries *et al*, 2004), while the incidence among black men has only recently begun to rise (McGlynn *et al*, 2003). The reason for this pronounced ethnic difference in risk is unknown.

Both molecular and epidemiologic studies have provided evidence that malignant transformation of testicular germ-cells occurs during early development (Rorth *et al*, 2000). It has been suggested that carcinoma *in situ* of the testis, a precursor of TGCT, has its origins in fetal life (Rorth *et al*, 2000) and that subnormal androgen exposure and/or increased oestrogen exposure are potentially important risk factors (Skakkebaek *et al*, 2001). Moreover, it is hypothesised that a disturbance in the fetal programming of gonadal development resulting from an intrauterine hormonal imbalance may delay differentiation of germ cells and render them more susceptible to malignant transformation (Rajpert-De Meyts *et al*, 1998).

A well-known hypothesis concerning TGCT has postulated that increased oestrogenic exposure during fetal life is associated with risk (Henderson *et al*, 1979; Sharpe and Skakkebaek, 1993). While some studies have supported this hypothesis (Moller and Skakkebaek, 1997; Sabroe and Olsen, 1998), two direct examinations of maternal hormone levels have not (Henderson *et al*, 1988; Troisi *et al*, 2003a). The direct examinations may have been hampered, however, by somewhat limited sample sizes and by studying samples drawn at only one time in pregnancy.

We have compared maternal serum levels of estradiol (total, free and bioavailable), estriol, sex hormone binding globulin (SHBG), testosterone (total, free and bioavailable) and alphafetoprotein during first and third trimesters in black and white populations with contrasting risks of TGCT.

## METHODS

### Study population

In order to examine the hypotheses of interest, a retrospective study of 150 pairs of black and white mothers was conducted among the participants of the United States Collaborative Perinatal Project (CPP). The CPP was a cohort study originally designed to examine perinatal risk factors for neurologic disorders in offspring (Niswander and Gordon, 1972). The study enrolled 41 796 pregnant women seen at 12 medical centres (Baltimore, Boston, Buffalo, Memphis, Minneapolis, New Orleans, New York (two centres), Philadelphia, Portland, Providence, and Richmond) in the United States between 1959 and 1965. Some women had more than one pregnancy during the study, resulting in approximately 55 000 births, of which 13 248 were white boys and 13 109 were black boys. The children born during the study were followed up until age 7 years. The mothers were asked to donate nonfasting blood samples at approximately 8-week intervals throughout their pregnancies. Serum samples have been stored in glass vials at −20°C with no recorded thaws.

Based on previous reports of differences in maternal hormone levels, power calculations determined that it would be necessary to examine samples from 150 white and 150 black mothers. Mothers were selected for inclusion based on characteristics of both the mother and the baby. Inclusion criteria based on characteristics of the mother were: first pregnancy, length of gestation between 26 and 48 weeks, and availability of blood samples from both first and third trimesters. Inclusion criteria based on characteristics of the baby were: male gender, singleton birth, birthweight equal to or greater than 500 g, baby lived for at least 1 year, and had no diagnoses of undescended testes, late descending testes, retractile testes or other malformations possibly related to maternal hormone levels (i.e., CNS and related musculoskeletal, genitourinary, inguinal hernia, hydrocele, supernumerary nipples).

A total of 162 black and 652 white mothers satisfied the study inclusion criteria. The principal limiting criterion was the availability of first trimester samples as the median entry time into the study for the whole CPP population was 20 weeks gestation. In addition, the nulliparity criterion restricted the study group to approximately one-third of the whole population. Each of the 162 black mothers was matched to a white mother on the closest blood draw dates. The matches were then reordered to select the 150 pairs best matched on draw dates.

### Laboratory assays

All hormone levels were assessed at the Reproductive Endocrine Research Laboratory of the University of Southern California Keck School of Medicine. Estriol, estradiol, and testosterone were determined by well-established and validated radioimmunoassay methods that are carried out routinely in the laboratory (Katagiri *et al*, 1974; Goebelsmann *et al*, 1979). Estriol was first extracted with hexane : ethyl acetate (10 : 1) to remove estrone and estradiol. Estriol in the aqueous fraction was then extracted with hexane :ethyl acetate (3 : 2). A separate serum sample was used to extract testosterone and estradiol, using ethyl acetate : hexane (3 : 2). This was followed by isolation of the two steroids using Celite column partition chromatography using ethylene glycol as the stationary phase. Testosterone and estradiol were eluted with 15% ethyl acetate in isooctane and 40% ethyl acetate in isooctane, respectively. Appropriate tritiated internal standards were added to each serum sample, prior to organic solvent extraction, to follow procedural losses. After drying each fraction, the estriol fraction was reconstituted in ethanol and the other fractions in assay buffer. An aliquot of each was taken to determine procedural loss, and duplicate aliquots were taken for the RIA of each hormone. Each RIA utilises an iodinated radioligand in conjunction with a specific antiserum (Diagnostic Systems Laboratories (DSL), Webster, TX, USA). A 7-point standard was included in each assay. After an overnight incubation (16–18 h), antibody-bound steroid was separated from unbound steroid by precipitation of the first antibody with a second antibody (DSL), and subsequent centrifugation.

Sex hormone binding globulin was quantified by direct chemiluminescent immunoassay using the Immulite analyzer (Diagnostic Products Corporation, Inglewood, CA, USA). The SHBG concentration and an average albumin concentration of pregnant women were then utilised in a validated algorithm with total testosterone and total estradiol, respectively, to calculate free testosterone; free- and albumin-bound testosterone (bioavailable testosterone); free estradiol; and free- and albumin-bound estradiol (bioavailable estradiol) (Sodergard *et al*, 1982).

Alphafetoprotein was quantified in serum by a solid-phase, two-site sequential chemiluminescent immunometric assay on the Immulite Analyzer (Diagnostic Products Corporation, Inglewood, CA, USA). The calibration range was up to 300 IU ml^−1^, using the WHO 1st International Standard 72/225. The analytical sensitivity of the assay was 0.2 IU ml^−1^. Various dilutions of samples (*N*=3) with high AFP levels demonstrated linearity, with observed/expected values ranging from 97 to 114%. Evaluation of assay specificity showed nondetectable crossreactivity with a variety of relevant proteins, for example, albumin, haemoglobin, transferrin, IgG.

A total of 68 samples from a single blood pool was assayed for quality control purposes. Each sample was assigned a unique, random identification number in order to blind subject identities. Samples were analysed in a total of 17 batches with each batch containing four quality control samples. The intra-assay coefficients of variation ranged between 3.7 and 12.6% for all hormones, while the inter-assay coefficients of variation ranged between 4.8 and 13.3%.

### Statistical analysis

Ratios of sex hormones were calculated using serum levels of total, free, and albumin-bound estradiol divided by serum levels of total, free, and albumin-bound testosterone, respectively. The ratios and serum hormone levels were logarithmically transformed to achieve approximately normal distributions and the transformed values were used in all statistical analyses. The geometric means of the hormone levels are presented. *T*-tests were employed for the mean comparison of potential confounding variables. Unmatched analysis of covariance (ANCOVA) was first used to compare mean log hormone levels and mean log hormone ratios between black and white mothers by trimester (first and third trimester), adjusting only for gestational age at blood draw. In subsequent analyses, adjustment was also made for maternal age, socioeconomic index, body weight (prepregnancy weight for first trimester and weight at delivery for third trimester) as continuous variables, and smoking status at registration as a categorical variable. Maternal age and socioeconomic index were included because they differed significantly between the white and black mothers. Although maternal weight and smoking status at registration did not differ significantly, adjusting for them resulted in changes of varying magnitude in the mean log hormone levels. Thus, they were also included in the final models. Pair-matched ANCOVA was used to test for differences between first and third trimester for each ethnic group. The statistical models for this matched analysis included an intercept for each mother, which adjusts for mother-level covariates. The homogeneity of between-trimester differences between the racial groups were determined using matched ANCOVA models by testing for an interaction between trimester and ethnicity after adjusting for the covariates that varied by trimester (i.e., gestational age at blood draw and body weight). Statistical analyses for this study were performed using the SAS system, version 8.02 (SAS Institute, Inc., Cary, NC, USA).

## RESULTS

A comparison of demographic characteristics of the participants found that the black mothers were younger and of lower socioeconomic status than the white mothers (Table 1). No significant differences in age at menarche, maternal height, prepregnancy weight, weight at delivery, or smoking were observed.

Hormone levels were first examined after adjusting solely for gestational ages at blood draw (Table 2). There were no significant differences between the black and white mothers’ levels of estradiols or estriol in first trimester. In third trimester, however, white mothers had significantly lower levels of SHBG, which resulted in their having significantly higher levels of free and bioavailable (free plus albumin-bound) estradiol. In addition, all testosterone levels (total, free, and bioavailable) and AFP levels were significantly higher among black mothers in both first and third trimesters. The ratios of testosterones to estradiols were also significantly different at both times, with black mothers having lower ratios than white mothers.

All hormone levels were re-examined after adjustment for maternal age, smoking status, socioeconomic status, and maternal weight, in addition to gestational ages at blood draw (Table 2). The adjustment affected both the estradiol and the SHBG findings. After adjustment, black mothers had significantly higher levels of total estradiol in first trimester (*P*=0.05), but in third trimester, there was no difference between any of the estradiol levels, or in SHBG levels. Similar to the unadjusted results, estriol levels did not vary between the black and white mothers at either time. Also in agreement with the unadjusted results, black mothers’ levels of all testosterones and of AFP remained significantly higher than white mothers’ levels and black mothers’ estradiol/testosterone ratios remained significantly lower than the ratios among white mothers at both time points.

Except for free and bioavailable testosterone, all other hormone levels and hormone ratios increased significantly from the first trimester to the third trimester in both white and black mothers (Table 3). The increases, however, were significantly greater among white mothers than black mothers for free estradiol, bioavailable estradiol, AFP, and for the hormone ratios. No significant difference was observed for the change in levels among the other measured hormones between white and black mothers. With the exception of AFP, all other hormones between first and third trimesters were significantly correlated in both black and white mothers (Table 4).

## DISCUSSION

It has long been suggested that exposure to maternal oestrogens is associated with TGCT (Henderson *et al*, 1979; Sharpe and Skakkebaek, 1993). Three potential mechanisms have since been postulated: (1) suppression of androgen production, (2) suppression of androgen receptor expression, and (3) suppression of secretion of insulin-like hormone 3 (Insl3) (Sharpe, 2003). It is clear that the first two mechanisms are directly associated with reduced androgen exposure. Insl3 is responsible for the transabdominal phase of testicular descent during first trimester through virilisation and outgrowth of the embryonic gubernaculum (Nef and Parada, 1999). Exposure to maternal oestrogens, including 17*α*- and *β*-estradiol, as well as diethylstilbestrol, downregulates Insl3 expression in embryonic Leydig cells (Emmen *et al*, 2000; Nef *et al*, 2000). On the other hand, based on the finding that oestrogens negatively regulate Leydig cell development via inhibition of replication of Leydig-cell precursor cells in adult and prepubertal rats, it is suggested that this regulatory mechanism may also control the number of Leydig cells in fetal life in humans (Sharpe and Skakkebaek, 1993). As Leydig cells are the source of testosterone and Insl3, if the above pathway is correct, the reduced number of Leydig cells resulting from the excess of oestrogen would decrease the production of testosterone and Insl3. Thus, elevated oestrogens during early pregnancy would, in theory, not only reduce the Insl3 expression but might also decrease testosterone production, and thus, result in an increased risk of TGCT. It should be noted, however, that the majority of undescended testes are located in the inguinal canal, rather than in the abdomen, suggesting a failure in the second, androgen-dependent, phase of descent rather than in the Insl3-dependent first phase. A more androgenic milieu during third trimester, then, may favour descent, in contrast with a less androgenic milieu.

Ethnic differences in maternal oestrogen and androgen levels have been reported by several epidemiologic studies. Also examining mothers in the CPP, Henderson *et al* (1988) studied first timester serum samples from 20 white and 20 black women and found no significant differences in levels of estradiol (total and free), SHBG or human chorionic gonadotropin. Contrary to expectation, the levels of total estradiol and free estradiol were higher in the black mothers (mean total estradiol=189.4 pg dl^−1^; mean free estradiol=1.66 pg dl^−1^) than the white mothers (mean total estradiol=138.4 pg dl^−1^; mean free estradiol=1.28 pg dl^−1^). The authors also reported that the black mothers had significantly higher levels of testosterone (114.4 ng dl^−1^) than did the white mothers (77.3 ng dl^−1^). Serum samples drawn from women at term, also showed no significant variation in levels of estradiol, estriol, or estrone between 50 white and 34 black mothers (Troisi *et al*, 2003a). As with the Henderson *et al* study, however, the authors reported that the black mothers had significantly higher testosterone levels than did white mothers. A similar lack of support for the oestrogen hypothesis was recently reported in a study comparing Chinese pregnant women to US white pregnant women. The Chinese women had significantly higher serum levels of estradiol and estriol at weeks 16 and 27 of gestation than did US white women (Hsieh *et al*, 2002). Chinese men, however, have a much lower incidence rate of TGCT than do US white men.

Our results are in agreement with these previously reported findings. We found significantly higher testosterone levels among black women in contrast with white women as did other studies: (Henderson *et al*, 1988; Troisi *et al*, 2003a). We also found higher total estradiol among black women than white women, which is in accord with other results (Henderson *et al*, 1988; Hsieh *et al*, 2002). If anything, these results may suggest that increased estradiol levels in first trimester are associated with reduced risk of TGCT, rather than increased risk. The *P*-value associated with the estradiol finding, however, just attained statistical significance and was unadjusted for multiple comparisons. An alternative explanation is that increased testosterone levels are associated with reduced risk of TGCT. Perhaps, however, it is not the absolute level of hormones that is critical, but the ratio of oestrogen to androgen. In the current study, black mothers had significantly lower ratios of sex hormones (oestrogens/androgens) than did white mothers during both the first and third trimesters. A lower ratio of total estradiol/total testosterone among black mothers has also been reported by others (Henderson *et al*, 1988).

Several studies have reported an association between maternal estriol and birthweight (Mucci *et al*, 2003). Parallel associations between increased birthweight and subsequent breast cancer risk have suggested that increased maternal estriol is a risk factor for breast cancer (Ahlgren *et al*, 2003). In contrast with breast cancer, most studies of birthweight and TGCT have reported that decreased birthweight, rather than increased birthweight, is a risk factor (Coupland *et al*, 2004), suggesting that maternal estriol levels might be lower in high-risk populations. The current study found some evidence to support this postulate. While there was no significant difference in estriol levels by ethnicity, black mothers did have higher estriol levels in third trimester than did white mothers as previously reported by Hsieh *et al* (2002), who found that second and third trimester estriol levels were significantly higher in Chinese mothers than in US white mothers.

A number of prior studies have examined ethnic differences in maternal serum AFP levels in second trimester. With some exceptions (Macri *et al*, 1976; Milunsky *et al*, 1980), the majority of studies have reported a higher level of weight-adjusted AFP among black mothers than white mothers (Crandall *et al*, 1983; Johnson, 1985; Baumgarten, 1986; Macri *et al*, 1987; Drugan *et al*, 1993; Benn *et al*, 1997; O’Brien *et al*, 1997). Ethnic differences in AFP levels during third trimester, however, have not been thoroughly studied. In the current research, weight-adjusted AFP levels were significantly higher among black mothers in both first and third trimesters. There was not, however, a significant correlation in either black or white mothers between first and third trimester levels of AFP. This lack of correlation may indicate that events during pregnancy influence AFP level more than they do levels of oestrogens and androgens, as the vast majority of AFP is produced by the fetus.

The current study has some strengths as well as some weaknesses. A major strength was the relatively large sample size, which granted sufficient statistical power to test the study hypothesis in both first and third trimesters. Another strength is that mothers of babies with conditions associated with aberrant hormone levels were excluded from the study. In addition, matching on the closest gestational age at blood draw for both first and third trimester decreased the likelihood of confounding by gestational age. A potential weakness of the study was the fact that the hormone levels were measured on samples that had been stored for roughly 40 years. Although long-term storage might have affected the hormone levels somewhat, it is unlikely that the bias is great as the levels in the current study are equivalent to levels reported in other studies examining newly drawn samples (Lipworth *et al*, 1999). A final weakness of the study is that it examined maternal hormone levels rather than fetal hormone levels and may not have been an accurate approximation of the *in utero* hormonal milieu. Comparisons of maternal and umbilical cord hormone levels found little correlation between the levels at term (Troisi *et al*, 2003b). Whether there is any correlation between maternal and fetal levels is not presently known and is unlikely to be examined until hormone assays on very small amounts of blood can be perfected. Given the uncertainty of the maternal–fetal correlations at the present time, the results of this, and other studies based on maternal levels, should be accepted with some caution.

In conclusion, the current study found little evidence to support the hypothesis that increased maternal oestrogen levels are associated with increased risk of TGCT. The data are consistent with several other hypotheses, however. Increased estradiol in first trimester may be associated with decreased risk of TGCT. Increased testosterone and/or increased AFP throughout pregnancy may be associated with decreased risk of TGCT, although a relationship with AFP seems somewhat unlikely. Decreased ratios of oestrogens/androgens throughout pregnancy may be associated with decreased risk of TGCT. Finally, an increased risk of estriol in third trimester may be associated with decreased risk of TGCT.

## Figures and Tables

**Table 1:** Selected characteristics of white and black mothers

	**White mothers (*n*=150)**		**Black mothers (*n*=150)**		
	**Mean**	**Standard deviation**	**Mean**	**Standard deviation**	***t*-test *P*-value**
Age at pregnancy (years)	22.26	3.67	19.65	4.31	<0.01
Age at menarche (years)	12.43	1.44	12.58	1.56	0.40
Prepregnancy weight (lbs)	123.08	17.47	124.55	18.57	0.49
Weight at delivery (lbs)	144.70	18.46	148.68	20.21	0.08
Height (inches)	63.90	2.42	63.55	2.64	0.23
Socioeconomic status index	73.23	17.13	41.29	23.82	<0.01

*Smoking at registration*					
No^a^	96 (0.64)		107 (0.71)		
Yes^a^	54 (0.36)		43 (0.29)		
Cigarettes per day	16.74	17.86	11.79	18.91	0.19

a Presented as numbers and proportion.

**Table 2:** Serum hormone concentrations between white and black mothers

	**White mothers (*n*=150)**		**Black mothers (*n*=150)**		**White *vs* black**	
**Hormone**	**Mean^a^^,^^b^ (95% CI)**	**Mean^a^^,^^c^ (95% CI)**	**Mean^a^^,^^b^ (95% CI)**	**Mean^a^^,^^c^ (95% CI)**	***P* b**	***P* c**
**First trimester^d^**						
Alphafetoprotein (IU ml^−1^)	6.9 (6.1–7.8)	7.0 (6.1–8.1)	10.7 (9.5–12.2)	10.5 (9.1–12.0)	<0.01	<0.01

Total estradiol (ng ml^−1^)	2.3 (2.1–2.6)	2.2 (2.0–2.5)	2.5 (2.3–2.8)	2.6 (2.4–2.9)	0.26	0.05
Free estradiol (pg ml^−1^)	24.6 (22.7–26.6)	23.5 (21.5–25.7)	24.8 (22.9–26.8)	25.9 (23.7–28.3)	0.89	0.17
Free and albumin-bound	534.7 (494.3–578.5)	511.8 (468.6–559.0)	538.6 (497.9–582.7)	562.7 (515.2–614.6)	0.90	0.18
Estradiol (pg ml^−1^)						

Total estriol (ng ml^−1^)	0.8 (0.7–0.9)	0.8 (0.7–0.9)	0.9 (0.8–0.9)	0.8 (0.7–0.9)	0.28	0.54

Total testosterone (ng ml^−1^)	1.0 (0.9–1.0)	1.0 (0.9–1.1)	1.5 (1.4–1.6)	1.4 (1.3–1.6)	<0.01	<0.01
Free testosterone (pg ml^−1^)	6.1 (5.6–6.6)	6.2 (5.7–6.7)	8.5 (7.9–9.2)	8.4 (7.7–9.1)	<0.01	<0.01
Free and albumin-bound testosterone (ng dl^−1^)	12.8 (11.8–13.8)	13.0 (12.0–14.2)	17.9 (16.6–19.3)	17.5 (16.1–19.1)	<0.01	<0.01

Sex hormone binding globulin (nmol l^−1^)	233.1 (217.1–250.2)	231.3 (213.7–250.3)	252.8 (235.5–271.3)	254.7 (235.4–275.7)	0.11	0.12

*Ratios*						
Total estradiol/total testosterone	2.4 (2.2–2.6)	2.2 (2.0–2.5)	1.7 (1.6–1.9)	1.8 (1.6–2.0)	<0.01	0.01
Free estradiol/free testosterone	4.1 (3.7–4.5)	3.8 (3.4–4.2)	2.9 (2.6–3.2)	3.1 (2.8–3.5)	<0.01	0.02
Free and albumin-bound estradiol/free and albumin-bound testosterone	41.9 (38.0–46.3)	39.3 (35.2–43.9)	30.1 (27.2–33.2)	32.1 (28.7–35.8)	<0.01	0.02

**Third trimester^e^**						
Alphafetoprotein (IU ml^−1^)	189.5 (175.3–204.9)	180.3 (165.6–196.3)	207.9 (192.3–224.8)	218.5 (200.7–237.8)	0.10	<0.01
Total estradiol (ng ml^−1^)	19.2 (17.9–20.5)	19.2 (17.8–20.7)	18.5 (17.3–19.7)	18.4 (17.1–19.9)	0.44	0.51
Free estradiol (pg ml^−1^)	119.5 (111.5–128.1)	117.2 (108.3–126.7)	106.3 (99.1–113.9)	108.4 (100.2–117.3)	0.02	0.21
Free and albumin-bound estradiol (pg ml^−1^)	2599.1 (2424.7–2786.0)	2547.5 (2355.3–2755.3)	2309.6 (2154.7–2475.7)	2356.5 (2178.7–2548.7)	0.02	0.21

Total estriol (ng ml^−1^)	11.2 (10.1–11.9)	11.7 (11.0–12.6)	11.3 (10.6–12.0)	10.8 (10.1–11.6)	0.83	0.13

Total testosterone (ng ml^−1^)	1.2 (1.1–1.3)	1.3 (1.2–1.4)	2.0 (1.8–2.1)	1.9 (1.7–2.0)	<0.01	<0.01
Free testosterone (pg ml^−1^)	1.3 (1.2–1.3)	1.3 (1.2–1.4)	1.7 (1.6–1.7)	1.6 (1.5–1.7)	<0.01	<0.01
Free and albumin-bound testosterone (ng dl^−1^)	2.0 (2.0–2.1)	2.1 (2.0–2.2)	2.4 (2.3–2.4)	2.4 (2.3–2.5)	<0.01	<0.01

Sex hormone binding globulin (nmol l^−1^)	503.2 (478.3–529.5)	514.4 (486.3–544.2)	544.7 (517.7–573.1)	532.9 (503.7–563.7)	0.03	0.43

*Ratios*						
Total estradiol/total testosterone	15.6 (14.4–16.9)	14.8 (13.5–16.2)	9.4 (8.7–10.2)	9.9 (9.1–10.9)	<0.01	<0.01
Free estradiol/free testosterone	31.2 (28.7–34.0)	29.5 (26.9–32.5)	18.6 (17.1–20.3)	19.6 (17.9–21.6)	<0.01	<0.01
Free and albumin-bound estradiol/free and albumin-bound testosterone	322.9 (296.7–351.5)	305.8 (278.3–336.0)	192.5 (176.9–209.6)	203.3 (185.0–223.4)	<0.01	<0.01

a Values are geometric means.

b Adjusted for gestational age of blood draw (continuous).

c Adjusted for maternal age at pregnancy (continuous), smoking at registration (ever/never), social economic index (continuous), gestational age of blood draw (continuous), and maternal weight (continuous).

d Adjusted maternal weight was prepregnancy weight.

e Adjusted maternal weight was weight at delivery.

**Table 3:** Changes of hormone levels between first and third trimesters by racial groups and the significance of the changes between racial groups^a^

	**White mothers (*n*=150)**		**Black mothers (*n*=150)**		**White mothers *vs* black mothers**
**Hormones**	**Mean^b^**	***P*-value**	**Mean^b^**	***P*-value**	***P*-value^c^**
Alphafetoprotein	3.3	<0.01	3.0	<0.01	<0.01

Total estradiol	2.1	<0.01	2.0	<0.01	0.11
Free estradiol	1.6	<0.01	1.5	<0.01	0.02
Free and albumin-bound estradiol	1.6	<0.01	1.5	<0.01	0.02

Total estriol	2.7	<0.01	2.6	<0.01	0.30

Total testosterone	0.2	<0.01	0.3	<0.01	0.36
Free testosterone	−0.5	<0.01	−0.4	<0.01	0.36
Free and albumin-bound testosterone	−0.5	<0.01	−0.4	<0.01	0.36

Sex hormone binding globulin	0.8	<0.01	0.8	<0.01	0.98

*Ratios*					
Total estradiol/total testosterone	1.9	<0.01	1.7	<0.01	0.01
Free estradiol/free testosterone	2.1	<0.01	1.9	<0.01	0.01
Free and albumin-bound estradiol/free albumin-bound testosterone	2.1	<0.01	1.9	<0.01	0.01

a Matched analyses.

b Means of the differences of the natural logarithm of hormone levels.

c Adjusted for gestational age at blood draw (continuous) and maternal weight at pre-pregnancy and delivery (continuous).

**Table 4:** Correlations of hormone levels between first and third trimesters by racial groups

	**White mothers (*n*=150)**		**Black mothers (*n*=150)**	
**Hormones**	**Pearson's correlation coefficient**	***P*-value**	**Pearson's correlation coefficient**	***P*-value**
Alphafetoprotein	−0.07	0.40	−0.02	0.77

Total estradiol	0.25	<0.01	0.35	<0.01
Free estradiol	0.41	<0.01	0.49	<0.01
Free and albumin-bound estradiol	0.41	<0.01	0.49	<0.01

Total estriol	0.16	0.05	0.43	<0.01

Total testosterone	0.53	<0.01	0.67	<0.01
Free testosterone	0.57	<0.01	0.56	<0.01
Free and albumin-bound testosterone	0.57	<0.01	0.56	<0.01

Sex hormone binding globulin	0.23	<0.01	0.43	<0.01
